# Visualization analysis on the research topic and hotspot of online learning by using CiteSpace—Based on the Web of Science core collection (2004–2022)

**DOI:** 10.3389/fpsyg.2022.1059858

**Published:** 2022-12-20

**Authors:** Youhua Shen, Lehui Huang, Xueshi Wu

**Affiliations:** ^1^Physical Education College, Jiangxi Institute of Applied Science and Technology, Nanchang, Jiangxi Province, China; ^2^Faculty of Education, Jiangxi Science and Technology Normal University, Nanchang, Jiangxi Province, China

**Keywords:** online learning, CiteSpace, visualization, web of science, hotspot

## Abstract

The objective of this research is to establish a better understanding of the current landscape of online learning research and development. Data were collected from the Web of Science (including SCI-EXPANDED, SSCI, and AHCI), which contains articles published from 2004 to 2022. A total of 25,382 pieces of data were collected. The data were visualized and analyzed using Citespace. The results show that the USA, China, and England are the main research countries in online learning; the Open University, Nanyang Technology University, and Monash University are the main research institutions; and Hwang Ggo-Jen, Huang Yueh-Min, and Chen Nian-Shing were the lead researchers. Major research topics in the field of online learning include MOOCs, flipped classrooms, COVID-19, computer-supported collaborative learning, the technology acceptance model, communities of inquiry, and distance learning. Meanwhile, each topic includes some classical literature. Computers & Education, Educational Technology Research and Development, the Internet, and Higher Education are three highly cited journals. Research hotspots mainly include three types of terms: student-related, learning-system-related, and teacher-related. Finally, we proposed further study ideas for future paths.

## Introduction

The COVID-19 epidemic has put a strain on the global education system, forcing colleges and universities to rethink their methods of instruction by removing face-to-face interaction from the classroom (Tarhini et al., 2017). As a result of the widespread use of information and communication technology (ICT), higher education has shifted to online platforms and adapted virtual teaching to deliver online courses (Hodges et al., 2020; Wahab, 2020). In the COVID-19 epidemic, where social distance is seen as the next degree of normalcy, there is an increasing urge to substitute physical engagement with virtual interaction. To meet the situation of educational institution closures prompted by COVID-19, UNESCO suggested that educational institutions equip themselves with online learning resources (Crawford et al., 2020). Because of COVID-19, online learning has become more popular all over the world. This is shown by the fact that more money is being spent on online learning projects related to education and that newer technologies and methods are being used in the field to encourage interaction between students and teachers.

Online learning is thought to have a significant impact on students’ academic achievement (Tawafak et al., 2020). It has developed as a potent learning medium, particularly when applying the internet as a delivery method. Learner satisfaction has skyrocketed following the successful adoption of online learning systems (Al-Fraihat et al., 2020). It is possible for professors and teachers to design their lessons, implement them, and keep track of the progress of their students using online learning tools. Because of this, it is very important for schools to provide a learning environment that both encourages students to do well and gives them room to grow.

In light of this, a number of researchers have achieved advancements in the profiling of literature on online learning. In these studies, a variety of perspectives have been explored, including the contribution of MOOCs to students’ equity and social inclusion (Lambert, 2020); self-regulated learning in MOOCs (Alonso-Mencía et al., 2020); the impacts of flipped classrooms on students (Aburezeq, 2020; Colomo-Magaña et al., 2020; Jdaitawi, 2020; Tang et al., 2020); learning strategies in flipped classrooms (Ahmad Uzir et al., 2020; Sandrone et al., 2020); the indicators of acceptance of e-learning (Al-Adwan et al., 2013); and the extended model of the Technology Acceptance Model (Abdullah and Ward, 2016). There is a dearth of bibliometric quantitative analysis in this discipline because of the reliance on expert opinion in the majority of cases (e.g., analysis of the frequency of words, the authors, the citations, the co-citations, the co-occurrences). Despite the fact that there are a lot of books and articles about online learning, its overall structure is unknown to the best of our knowledge.

To overcome the subjectivity of research, knowledge mapping—a new multidisciplinary field of study that aims to track, mine, analyze, sort, and present information (Shiffrin and Borner, 2004)—is introduced into the field of online learning. CiteSpace, software that incorporates bibliometric analysis, data mining tools, and visualization techniques, is better at making visualizations clear and easy to understand than other tools. This makes it easier for users to identify the most essential trends and critical points without having to think too hard about it (Chen, 2006).

So far, only a small amount of research had been done with CiteSpace to analyse the rapidly expanding online learning literature. Based on the China National Knowledge Infrastructure (CNKI; 2000–2020), CiteSpace was used by Huang and Zhou (2021) to assess the knowledge map of online educational resources in the 21st century and examine the changes and development of digital learning resource research. Based on data from the Web of Science (WoS), Park and Shea (2020) conducted a co-citation study of online learning research trends over the past 10 years and discovered features and changes in research trends in online learning as well as the most cited publications in the field. Based on the WoS database from 1995 to 2018, Negahban and Zarifsanaiey (2020) investigated the research trends in the field of e-learning by assessing relevant studies employing network analysis and scientific mapping techniques and identified the most frequently used terminology, the greatest impact studies, and some topics that served as bridges to connect different topics. Based on the CNKI database from 2000 to 2021, Chi (2021) analyzed the number distribution of blended learning literature, core authors, and research institutions, and further conducted keyword co-occurrence analysis, emergence detection analysis, and cluster time domain mapping analysis by using the knowledge mapping tool CiteSpace. Based on the WoS dataset from 2012 to 2018, Zheng et al. (2019) mapped the knowledge mapping of MOOCs research and revealed the most important individuals and organizations, as well as well-known terms and periodicals in this field’s scholarly literature. The existing literature had laid a foundation for understanding and in-depth research in the field of online learning. However, there are still certain drawbacks, such as a lack of rigorous bibliometric analysis, limiting literature sources and low sample sizes.

To give a holistic and impartial overview of research on online learning, this study utilizes a scientometric analysis based on CiteSpace to identify bibliometric traits and show relationships among publications on this subject published in WoS journals between 2004 and 2022. More precisely, this study includes four research questions:(1) What are the characteristics of collaboration in the field of online education? (2) Who are the most highly cited researchers and journals in the field? (3) How do the primary areas of knowledge change over time? (4) What new subjects have emerged in recent years in the research of online learning?

## Materials and methods

### Research tools

Scientometric analysis is an important tool for dealing with data and information visualization, and it may be used to identify research frontiers and hotspots, as well as track key developments in one field. To prevent subjectivity, scientometric analysis primarily uses statistical methodologies to assess, analyze, and evaluate the quality and characteristics of research materials. According to a massive collection of publications in the database of scholarly literature, this approach is recognized as a popular tool for learning about a single topic, summarizing the development route, and forecasting the future trend. Many literature analysis programs, notably CiteSpace, are employed in scientometric analysis.

CiteSpace is a Java-based program developed by Chaomei Chen, a well-known academic whose research interests include information visualization, knowledge mapping, and scientific frontier atlases. CiteSpace is an effective tool for swiftly acquiring knowledge on a certain topic. CiteSpace’s underlying approach is to designate co-citation clusters and then create timeliness and critical spots using time-sliced snapshots. CiteSpace 6.1.R1 (64-bit) was utilized in this study, which runs on the Java 8 environment. Kleinberg’s burst detection, Freeman’s betweenness centrality (BC) measure, and heterogeneous networks are all included in the latest edition. This means that three critical issues may be addressed more effectively: (1) establishing research frontiers, (2) categorizing specializations, and (3) spotting developing trends and abrupt points. Moreover, the number of publications employing CiteSpace has expanded dramatically.^1^

### Data collection

Data gathering is the most important component of a review article, and it plays a pivotal role in determining the quality and effectiveness of a review article. The WoS is among the most famous citation indexes. Scientists can use the Science Citation Index (SCI), the Social Science Citation Index (SSCI), as well as the Arts and Humanities Citation Index (AHCI) to do their research (Fu et al., 2022; Wu L. and Wang, 2020). In order to discover the most relevant and appropriate articles, this article employed a certain procedure for collecting and selecting information: (1) Select the WoS Core Collection; (2) Choose the advanced search option; (3) abide by Table 1 search requirements; and (4) finalize the collection of data. After removing duplicates, a total of 25,382 qualified records were retained.

**Table 1:** Detailed search setting parameters.

Source	WoS core collection
Citation	SCI-EXPANDED,SSCI,AHCI
Search conditions	ts = (“digital education” OR “online learning” OR “Digital learning” OR “Electronic Learning” OR “Online-Merge-Offline” OR “distance teaching” OR “remote instruction” OR “distance learning” OR “remote education” OR “online teaching” OR “online education” OR “blended learning” OR “online reading” OR “smart learning” OR “e-learning” OR “massive open online courses” OR “online course” OR “Computer supported collaborative learning” OR “Immersive Learning” OR “smart education” OR “moodle” OR “Small Private Online Course” OR “Massive Private Online Course” OR “flipped classroom” OR “ubiquitous learning” OR “online course”) AND language:(English) Type: Article, Published Online, Review
Time span	2004–2022
Qualified records	25,382

### Research framework

**Figure 1:**
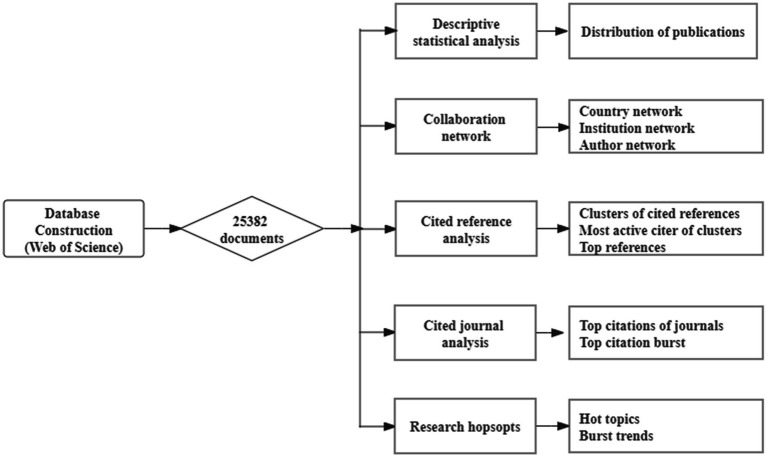
General framework of this study.

In this paper, we provide an integrated framework for interpreting the trends and changes in online learning for a total of 25,382 publications from 2004 to 2022 (Figure 1). Stable results can be achieved by tweaking the parameters over and over again. During this investigation, the following tasks were accomplished: (1) descriptive statistical analysis, including the distribution of publications, with the goal of gaining a comprehensive picture of this field; (2) collaboration network analysis, including three levels, namely nation, institution, and author, is intended to describe the main contributions of online learning from the macro, medium, and micro levels; (3) cited reference analysis, including cluster of cited references, most active citer of clusters, and top references, intended to identify primary research topics, and the classic literature; (4) cited journal analysis, including top citation of journals, and top citation burst; (5) research hotspots, analyzing the current research hotspots of online learning through keyword co-occurrence network analysis and keyword clustering, dividing hotspot keywords into three types, and analyzing the evolution of the research topic. Researchers have discovered five potential future research trajectories using burst detection.

## Results

### Research outputs

**Figure 2:**
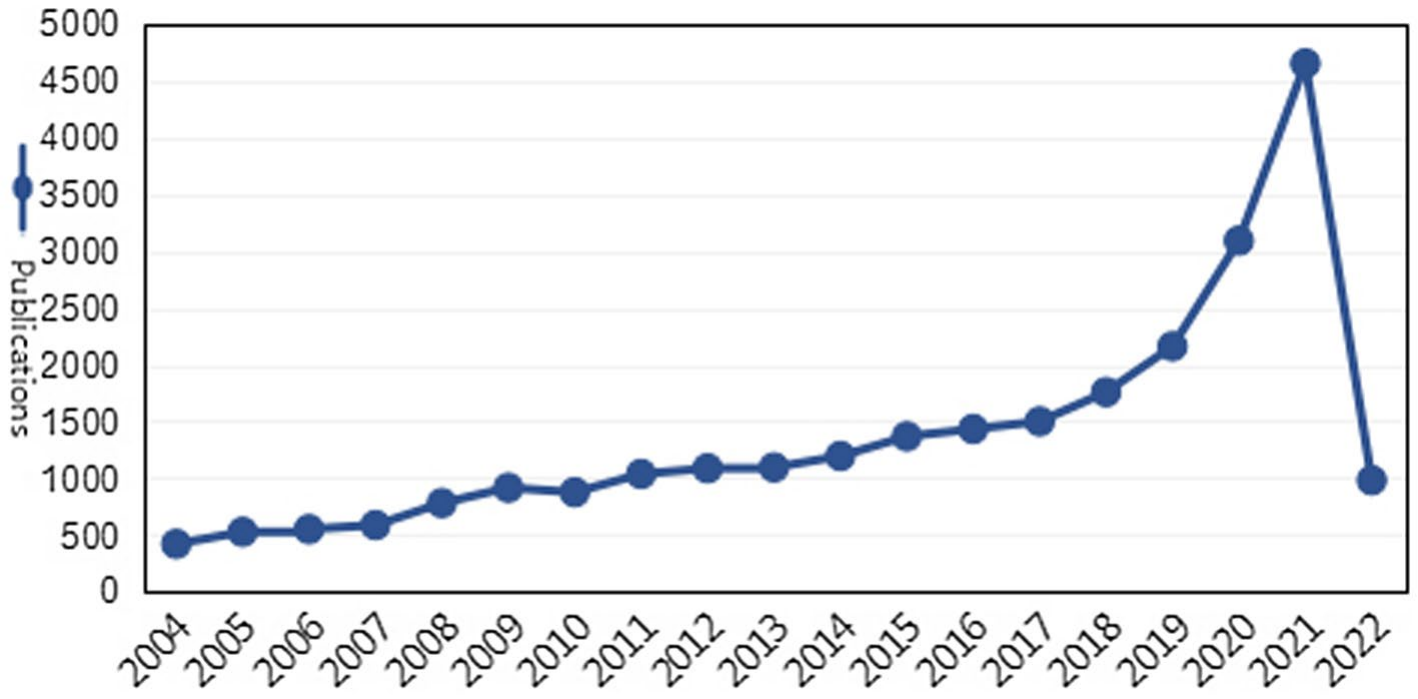
Publications from 2004 to 2022.

The progression of papers published related to online learning during the 19-year period 2004–2022 is shown in Figure 2. There has been a definite growing trend in the use of scientific research in online learning throughout the years. According to the growth curve of online learning research, three stages can be identified as follows:

(1) Stage of slow development (2004–2010). Prior to 2008, the number of papers published each year averaged around 500. Although researchers had recognized that online learning could be a useful complement to traditional learning, the main research content at this stage was mostly more general theoretical and practical explorations, such as barriers to online learning for students (Muilenburg and Berge, 2005), students’ experiences of online learning (Sit et al., 2005), learner characteristics and their approaches to managing learning (Del Valle and Duffy, 2009; Nakayama et al., 2012), and combining online learning with traditional methods (Condie and Livingston, 2010).

(2) Stage of rapid development (2011–2016). By this stage, some empirical studies on online learning had been produced. The research in this period mainly includes two categories: one is the influence of online learning on students (Chen et al., 2011; Keller and Karau, 2013; Xu and Jaggars, 2013; Shea and Bidjerano, 2014); the second is the factors affecting students’ online learning participation, performance, and satisfaction (Hu et al., 2014; Lehmann et al., 2014; Pellas, 2014). In comparison to previous years, the volume of articles has increased significantly faster now. The annual number of articles published exceeded 1,000 during this period.

(3) Stage of explosion (2017–2022). Since 2017, a growing number of policymakers, academics, and international organizations are taking a closer look at online learning difficulties. During this period, there were more than 1,500 articles published annually. The main reasons behind this may include two aspects: first, the rapid development of information and communication technology provides a foundation for the popularization of online learning (Hodges et al., 2020); second, during the COVID-19 pandemic, in order to avoid large-scale transmission of the virus, schools temporarily stopped face-to-face offline teaching and switched to online learning (Crawford et al., 2020). According to incomplete statistics, about 1.2 billion students have used online learning so far. At the same time, research on online learning has grown explosively.

### Collaboration network

#### Country collaboration network

**Figure 3:**
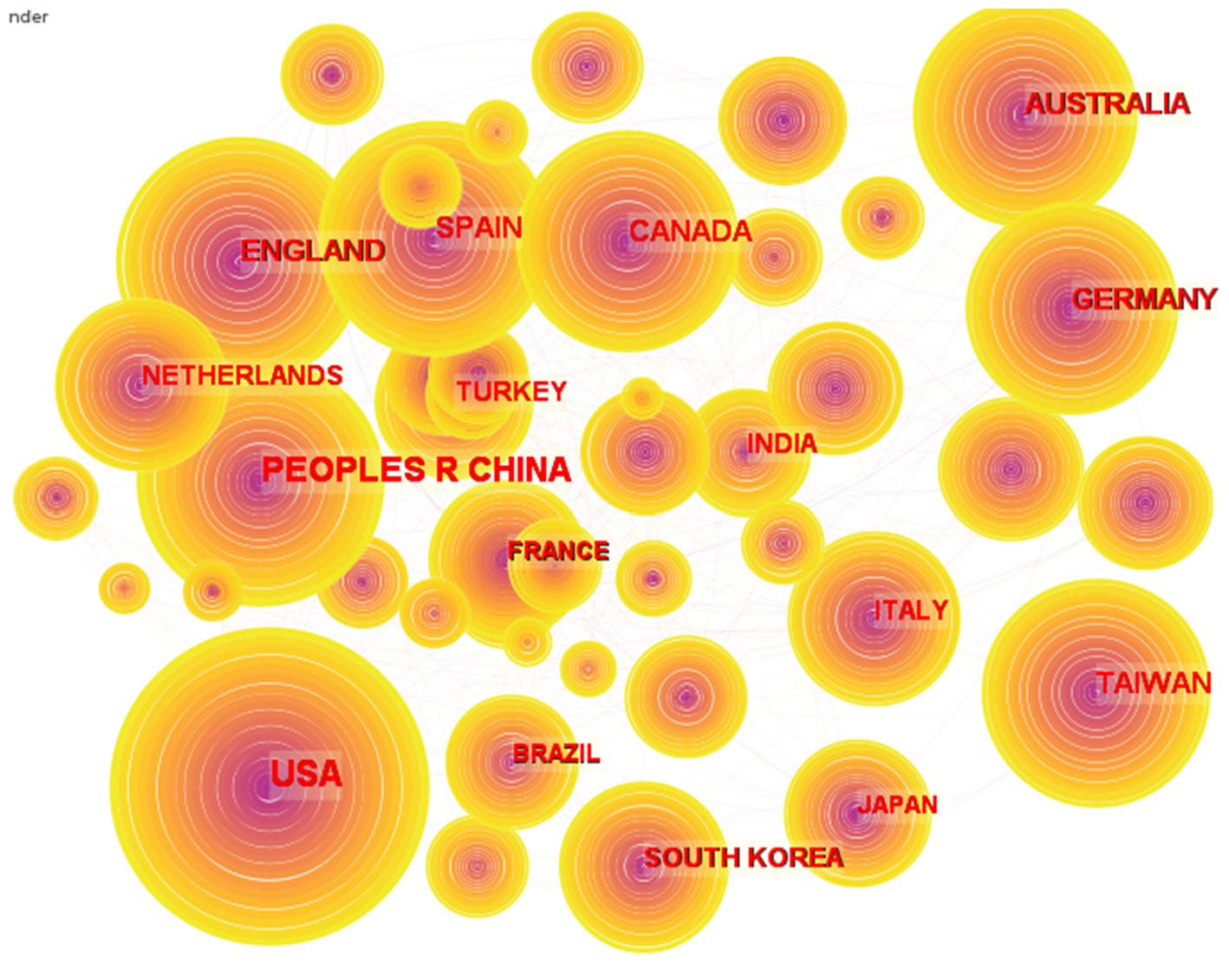
Visualization of the country collaboration network.

Figure 3 depicts the collaboration network, which had 243 nodes and 3,452 linkages between 2004 and 2022, and Table 2 lists the 10 nations that contributed the most to the overall outputs. The United States publishes the most papers (6,948), followed by China [4,978, including the Taiwan district (1,582)] as the second greatest provider. England ranked third, as its number of publications was 1,986. In general, the number of outputs is proportional to the number of research institutes and the amount of funds available for research. Another clear explanation for increased research output is that the unique coronavirus epidemic has prompted educational institutions in the great majority of nations to shift from conventional face-to-face instruction to online teaching. As a node’s BC indicates how many shortest routes flow through it, the node’s relevance grows. CiteSpace uses this statistic to assess a node’s importance to the network. An essential reference for the field is judged to have a betweenness value of 0.1 or more. It is clear that European countries play a big part in making connections with other countries because of their high BC, including Switzerland (0.12), Finland (0.12), and France (0.1).

**Table 2:** Top 10 countries based on frequency and BC.

Country	Frequency	Country	BC
USA	6,948	England	0.38
China	3,396	Canada	0.22
England	1986	Sweden	0.22
Spain	1921	France	0.18
Australia	1,685	India	0.13
Canada	1,262	Saudi Arabia	0.13
Germany	1,197	Scotland	0.13
Turkey	762	USA	0.12
Netherlands	686	Portugal	0.12
Portugal	632	Australia	0.11

#### Institution collaboration network

**Figure 4:**
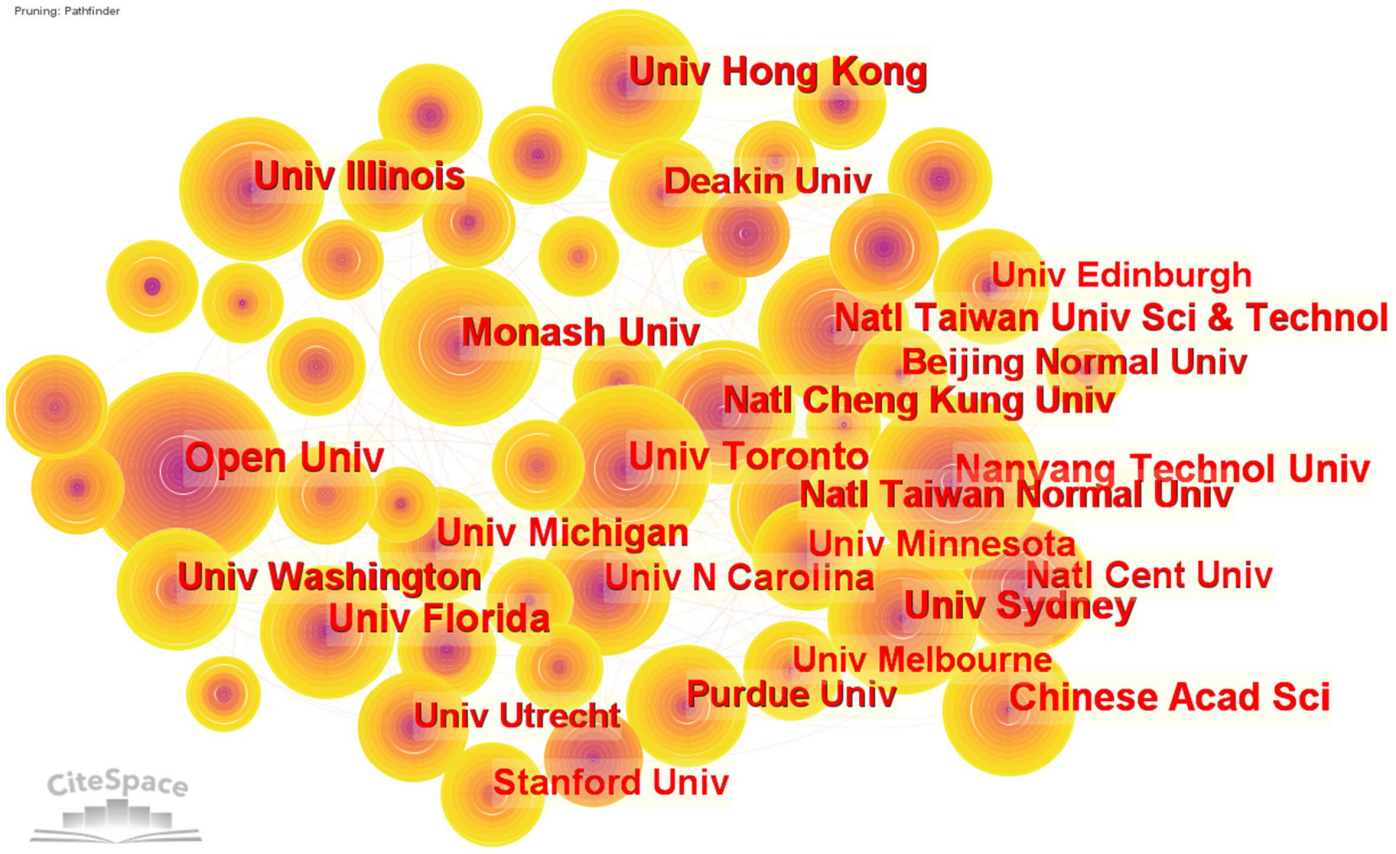
Visualization of the institution collaboration network.

Figure 4 depicts the collaboration network of universities between 2004 and 2022, which included 1,652 institutions and 3,985 linkages. Even if there are some connections between the nodes, the colors are lighter, indicating that although there is some collaboration between the nations, the degree of cooperation is not profound at this time. Because of this, there is greater room for growth in the area of online education in the future. Table 3 lists the top 20 institutions in terms of overall outputs and the percentage of their outputs that they contributed to. With 231 publications, the Open University takes the top spot on the list, and Nanyang Technological University (202), Monash University (186), the University of Toronto (180), and the University of Sydney (180) are additional institutions with a substantial number of publications (174). Clearly, institutional contributions to online learning line up with countries.

**Table 3:** A list of the top 20 most frequently visited institutions.

Institution	Frequency	Country
Open Univ	231	United Kingdom
Nanyang Technol Univ	202	Singapore
Monash Univ	186	Australia
Univ Toronto	180	Canada
Univ Sydney	174	Australia
Univ Hong Kong	169	China
Univ Illinois	155	America
Chinese Acad Sci	153	China
Univ Florida	149	America
Natl Cheng Kung Univ	143	Singapore
Natl Taiwan Normal Univ	142	China
Natl Taiwan Univ Sci & Technol	142	China
Univ Michigan	129	America
Univ Washington	126	America
Natl Cent Univ	124	China
Univ N Carolina	119	America
Beijing Normal Univ	117	China
Purdue Univ	115	America
Deakin Univ	113	Australia
Univ Minnesota	108	Australia

#### Author collaboration network

**Figure 5:**
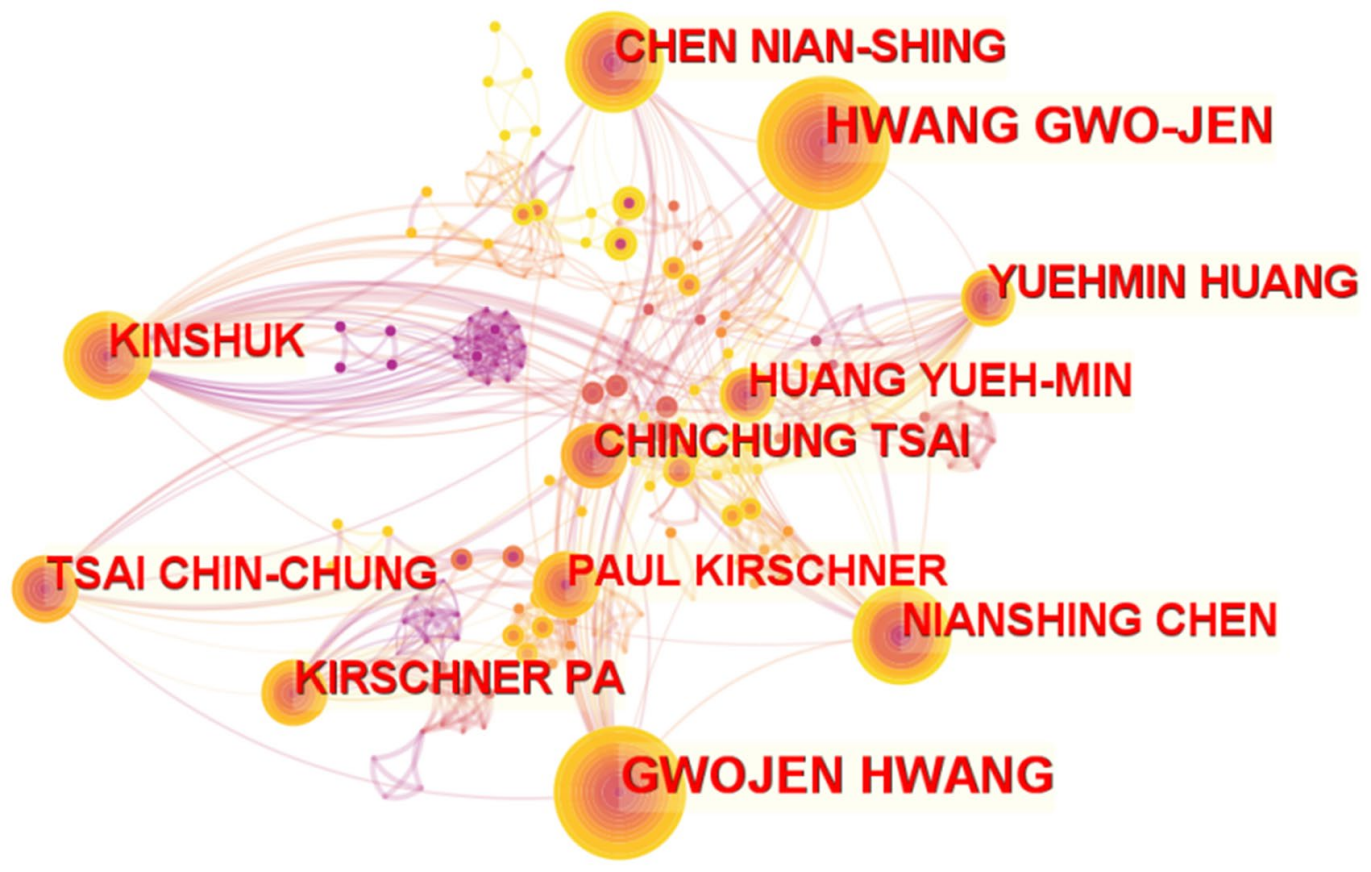
Visualization of the author network.

There were 3,765 authors and 9,142 cooperation ties in the author collaboration network for online learning research depicted in Figure 5. Research in online learning is multidisciplinary because of the network’s size and scope and the variety of collaborations among its members. HWANG GWO-JEN appears to be the most prolific author in the area of online learning; he has worked on the U-learning environment and learning strategy, intelligent online learning, and game-based learning strategy for over 15 years. Furthermore, three major authors (HWANG GWO-JEN, HUANG YUEH-MIN, CHEN NIAN-SHING) developed a closer relationship since 2008. Table 4 shows that the majority of the writers in the top 10 list are associated with a department or faculty that specializes in digital learning, engineering, information, or electronics.

**Table 4:** Top 10 authors based on frequency.

Frequency	Author	Institution
57	Hwang et al. (2008)	Graduate Institute of Digital Learning and Education, National Taiwan University of Science & Technology
39	Huang et al. (2006)	Department of Engineering Science, National Cheng Kung University
35	Chen et al. (2007)	Department of Information Management, National Sun Yat Sen University
35	Kirschner et al. (2004)	Research Centre for Learning, Teaching and Technology, Open University Netherlands
34	Kinshuk and Lin (2004)	College of Information, University of North Texas System
34	Tsai (2008)	Graduate Institute of Digital Learning and Education, National Taiwan University of Science & Technology
34	Schaar and Lin (2011)	Department of Electrical and Computer Engineering, University of California Los Angeles
31	Kirschner and Lai (2007)	Research Centre for Learning, Teaching and Techology, Open University Netherlands

### Knowledge structure map

The development of a new subject necessitates the accumulation of knowledge in related fields. Research papers cannot be generated purely based on their own content. The article should draw on previous research and literature in the field or in related fields. Journal articles are generally considered to represent the cutting edge of specific areas, and references in these articles are often used as a basis for further research. We were able to detect co-citation clusters using a computer application that let us locate common citations in online learning. Using journal articles to visualize online learning research’s foundational knowledge is a critical first step in identifying such information.

**Figure 6:**
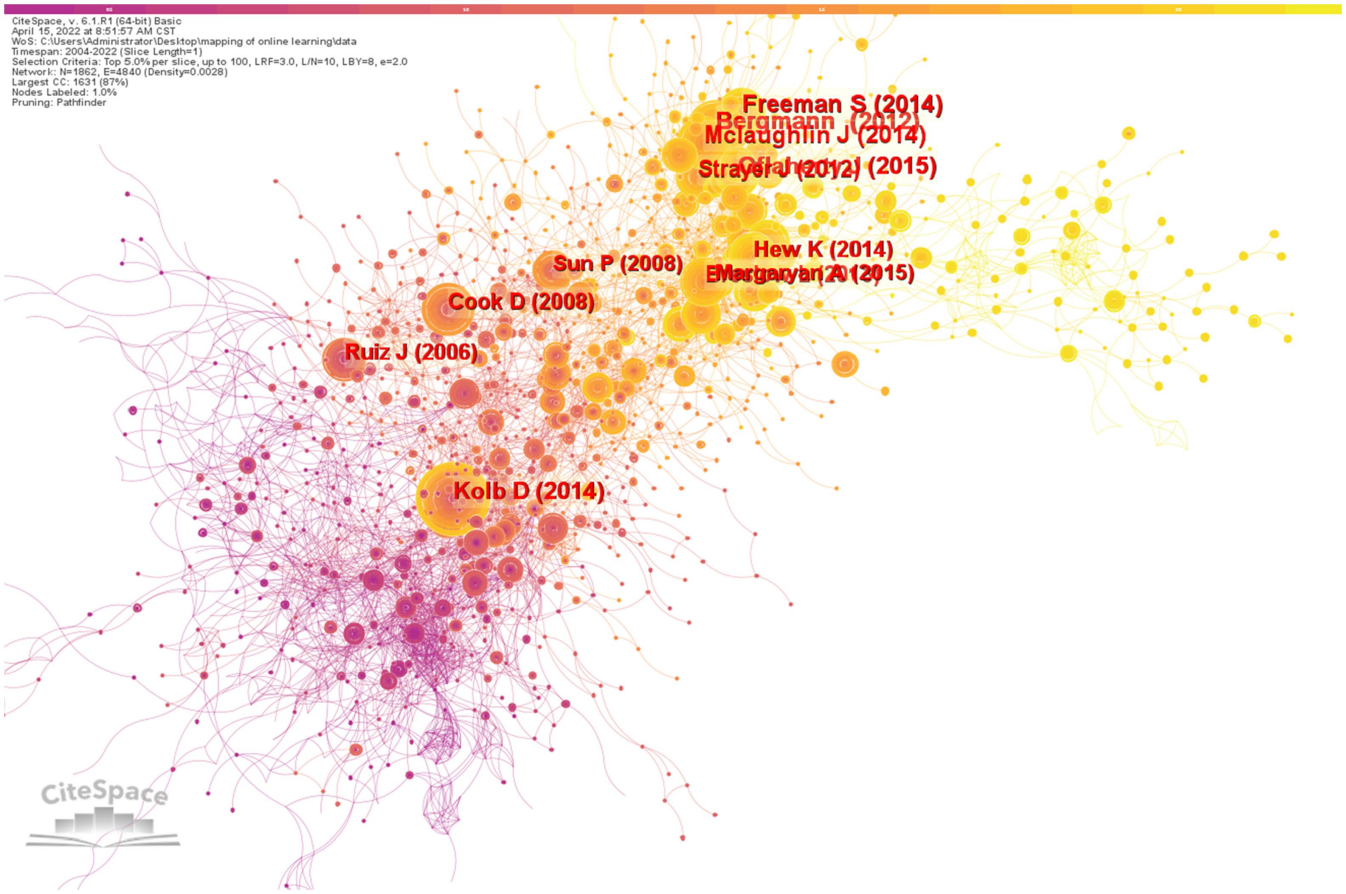
Mapping of cited reference.

**Figure 7:**
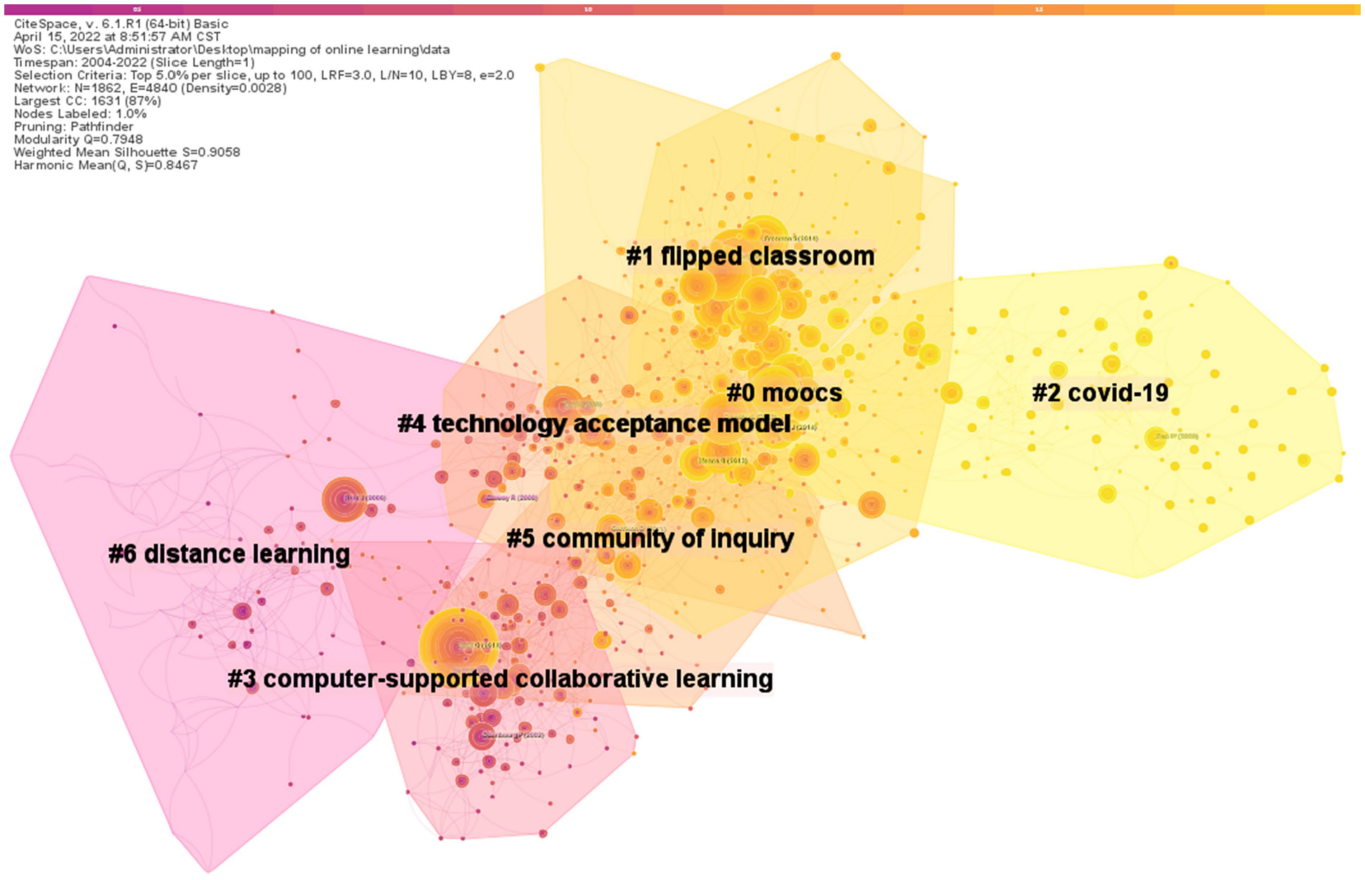
Co-citation clusters of cited references.

CiteSpace was configured with the parameters listed below: (1) Time slicing: from 2004 to 2022,and years per slice: 1; (2) Term source: title, abstract, descriptors, identifiers; (3) Node type: cited reference; (4) Pruning: pathfinder and pruning the merged network; (5) Top N per slice: Select top 50 most cited articles per slice. CiteSpace generated a map depicted in Figure 6. For the clustering, this study employed log-likelihood ratios and the labeling source of ‘T’. According to co-citation cluster statistics, there are 27 knowledge clusters, seven of which are large clusters (Figure 7).

Modularity Q ranges from 0 to 1, with values closer to 1 indicating tighter relationships and connections within the cluster. As a general rule, modularity Q levels between 0.4 and 0.8 can be deemed appropriate (Chen et al., 2014). The range for the Mean Silhouette is −1 to −1. Content consistency or similarity is high when a cluster’s value is close to 1 (Chen et al., 2014). According to Figure 7 and Table 5, Modularity Q is 0.7948, and the Mean Silhouette is 0.9058. The Silhouette values for each of the 27 clusters are more than 0.8. This means that the online learning research mapping has undergone a high-quality cluster analysis.

**Table 5:** Details of knowledge clusters.

ID	Size	Silhouette	Year	Label (LLR)
0	136	0.888	2017	**MOOCs** (100.05, 1.0E-4); mooc (91.27, 1.0E-4); massive open online courses (69.47, 1.0E-4); massive open online course (mooc) (30.15, 1.0E-4);
1	131	0.95	2017	**Flipped classroom** (302.26, 1.0E-4); active learning (66.96, 1.0E-4); flipped learning (56.35, 1.0E-4);
2	129	0.969	2021	**COVID-19** (266.26, 1.0E-4); covid-19 pandemic (47.92, 1.0E-4); pandemic (39.18, 1.0E-4);
3	121	0.858	2009	**Computer-supported collaborative learning** (59.73, 1.0E-4); collaborative learning (32.65, 1.0E-4);
4	114	0.858	2013	**Technology acceptance model** (22.88, 1.0E-4); e-learning (18.51, 1.0E-4); flipped classroom (16.28, 1.0E-4);
5	109	0.852	2013	**Community of inquiry** (49.5, 1.0E-4); social presence (38.98, 1.0E-4); teaching presence (29.6, 1.0E-4);
6	108	0.922	2005	**Distance learning** (25.48, 1.0E-4); internet (20.91, 1.0E-4); medical education (18.04, 1.0E-4); e-learning (13.74, 0.001);

Specifically, from Table 5, it can be seen that the biggest and most important cluster was MOOCS (#0). This cluster of studies focuses on motivations and challenges, instructional quality, enrolment and completion, and so on. A total of 136 items were found in this cluster, most of which were published in 2017. This cluster has a silhouette value of 0.888, indicating that the 136 cited literatures in the cluster had a high consistency, with Hew and Cheung's (2014) article on motivation and challenges of MOOCs use being referenced to most frequently (among this cluster, 42% of the articles cited Hew’s article, which was published in the Educational Research Review). The second most-cited article was Margaryan’s et al. (2015) on instructional quality, published in the Computers and Education and cited by 37% of the articles in the cluster. The essay by Liyanagunawardena et al. (2013) came in third place in terms of citations, which is a systematic literature review between 2008 and 2012. A 2014 study by Jordan published in the International Review of Research in Open and Distributed Learning, which investigated initial tendencies in enrolment and completion, was the article with the fourth-highest number of citations.

The second largest cluster, labelled “flipped classroom” (#1), contains 131 articles with a silhouette value of 0.95. O’Flaherty and Phillips’s (2015) paper on students’ satisfaction with blended instructional design was the most cited, with 41 percent of the 131 articles. The third largest cluster (#2) contains 129 articles with a silhouette value of 0.969, which indicates the high consistency of this cluster. The most-cited article within the cluster was Dhawan’s (Dhawan, 2020) article on a systematic literature review on the definition of online learning. Details about these clusters are shown in Table 6.

**Table 6:** Most active citer of the clusters.

Coverage	Author (year)	Articles
Cluster #0		
42%	Hew and Cheung (2014)	Students’ and instructors’ use of massive open online courses (MOOCs): Motivations and challenges
37%	Margaryan et al. (2015)	Instructional quality of Massive Open Online Courses
32%	Liyanagunawardena et al. (2013)	MOOCs: A systematic study of the published literature 2008–2012
32%	Jordan (2014)	Initial trends in enrolment and completion of massive open online courses
Cluster #1		
51%	O’Flaherty and Phillips (2015)	Students’ Satisfaction with a Blended Instructional Design: The Potential of” Flipped Classroom” in Higher Education.
41%	Strayer (2012)	How learning in an inverted classroom influences cooperation, innovation and task orientation
Cluster #2		
23%	Dhawan (2020)	How many ways can we define online learning? A systematic literature review of definitions of online learning (1988–2018)
22%	Rose (2020)	Medical student education in the time of COVID-19
21%	Bao (2020)	COVID-19 and online teaching in higher education: A case study of Peking University
21%	Henseler et al. (2015)	A new criterion for assessing discriminant validity in variance-based structural equation modeling
Cluster #3		
25%	De Wever et al. (2006)	Content analysis schemes to analyse transcripts of online asynchronous discussion groups: a review
18%	Kirschner et al. (2006)	An analysis of the failure of constructivist, discovery, problem-based, experiential, and inquiry-based teaching
17%	Weinberger et al. (2005)	Epistemic and social scripts in computer-supported collaborative learning
17%	Fischer et al. (2013)	Toward a script theory of guidance in computer-supported collaborative learning
Cluster #4		
45%	Sun et al. (2008)	What drives a successful e-Learning? An empirical investigation of the critical factors influencing learner satisfaction
22%	Liaw (2008)	Investigating students’ perceived satisfaction, behavioral intention, and effectiveness of e-learning: A case study of the Blackboard system
21%	Alraimi et al. (2015)	Understanding the MOOCs continuance: The role of openness and reputation

Analysis was also carried out to determine the most widely cited papers based on the amount of citations (Table 7). Top 5(citation above 600) most-cited studies include: O’Flaherty and Phillips’s (2015) study on the impact of flipped classroom on students’ learning satisfaction and involvement (citation 1,295); Sun et al. (2008)‘s study on the influencing factors of students’ satisfaction with e-learning (citation 1,033); Hew and Cheung (2014)’s study on motivation, challenges, and unresolved issues in MOOCs use (citation 879); Strayer (2012)‘s study comparing flipped classroom and traditional classroom (citation 190); Margaryan et al. (2015)’s study on the quality of MOOCs instructional design (citation 655).

**Table 7:** Top five references with cited frequency.

Authors	Articles	Cited frequency	Year
Oflaherty	Students’ Satisfaction with a Blended Instructional Design: The Potential of” Flipped Classroom” in Higher Education.	1,295	2015
Sun	What drives a successful e-Learning? An empirical investigation of the critical factors influencing learner satisfaction	1,033	2008
Hew	Students’ and instructors’ use of massive open online courses (MOOCs): Motivations and challenges	879	2014
Strayer	How learning in an inverted classroom influences cooperation, innovation and task orientation	790	2012
Margaryan	Instructional quality of massive open online courses (MOOCs).	655	2015

A surge in citations is a sign that a certain field of study is becoming more and more well-known. Evidence of a citation spike might be seen in the form of citation bursts (Chen et al., 2014). With a citation burst value of 23.96, Bergmann and Sams's (2012) paper from cluster #6 came out on top. Indicators such as an increase in citations to a single article during a period of strong activity in a particular field of study are known as a “citation burst” (Chen et al., 2014). Table 8 lists the top 20 burst-based authors and research.

**Table 8:** Top 20 references with strongest citation bursts.

References	Year	Strength	Begin	End
Palloff and Pratt, 1999, *Jossey-Bass,* CO, V0	1999	14.15	2004	2007
Ruiz et al., 2006, *Academic Medicine*, V81, P207	2006	22.37	2007	2014
Garrison and Kanuka, 2004, *The Internet and Higher Education*, V7, P95	2004	15.9	2007	2012
Bernard et al., 2004, *Review of Educational Research*, V74, P379	2004	13.74	2007	2012
Cook et al., 2008, *The Journal of the American Medical Association*, V300, P1181	2008	20.38	2009	2016
Sun et al., 2008, *Computers & Education*, V50, P1183	2008	17.97	2010	2016
Bergmann and Sams, 2012, *International Society for Technology in Education*, V0	2012	23.96	2015	2019
Mclaughlin et al., 2014, *Academic Medicine*, V89, P236	2014	18.71	2015	2020
Margaryan et al., 2015, *Computers & Education*, V80, P77	2015	13.53	2015	2020
Strayer, 2012, *Learning Environments Research*, V15, P171	2012	14.77	2016	2020
Davies et al., 2013, *Educational Technology Research and Development*, V61, P563	2013	13.41	2016	2020
Freeman et al., 2014, *Proceedings of the National Academy of Sciences*, V111, P8410	2014	20.8	2017	2022
Hew and Cheung, 2014, *Educational Research and Reviews*, V12, P45	2014	15.45	2017	2022
O’Flaherty and Phillips, 2015, *The Internet and Higher Education*, V25, P85	2015	18.78	2018	2020
Abeysekera and Dawson, 2015, *Higher Education Research & Development*, V34, P1	2015	18.7	2018	2020
Mason et al., 2013, *IEEE Transactions on Education*, V56, P430	2013	14.26	2018	2020
Hone and El Said, 2016, *Computers & Education*, V98, P157	2016	13.64	2019	2022
Kizilcec et al., 2017, *Computers & Education*, V104, P18	2017	13.5	2019	2022
Cao et al., 2020, *Psychiatry Research*, V287, P0	2020	18.24	2020	2022
Rose, 2020, *The Journal of the American Medical Association*, V323, P2131	2020	14.58	2020	2022

Purple rings indicate significant research with a high BC. A larger circle signifies a greater degree of importance in betweenness. Research with a centrality rating of equal to or more than 0.1 is generally regarded as an important study. For example, Means (1988)’s study on meta-analysis of online learning had a centrality value of 0.18. Rourke et al. (1999)’s study had a centrality value of 0.11. Table 9 specifies the top 9 key pieces of literature in the online learning knowledge map.

**Table 9:** Top nine references by centrality.

Centrality	References
0.18	Means et al., 2009, *Evaluation Evidence,* V0, P0
0.11	Rourke et al., 1999, *The Journal of Distance Education*, V14, P50
0.11	Pena-Shaff and Nicholls, 2004, *Computers & Education*, V42, P243
0.1	Kolb, 2014, *FT Press*, V0, P0
0.09	Broadbent and Poon, 2015, *The Internet and Higher Education*, V27, P1
0.08	Schellens and Valcke, 2006, *Computers & Education*, V46, P349
0.08	Tucker, 2012, *Education Next,* V12, P82
0.08	Schrire, 2006, *Computers& Education*, V46, P49
0.08	Garrison et al., 2001, *American Journal of distance education*, V15, P7

### Distribution of cited journals

**Figure 8:**
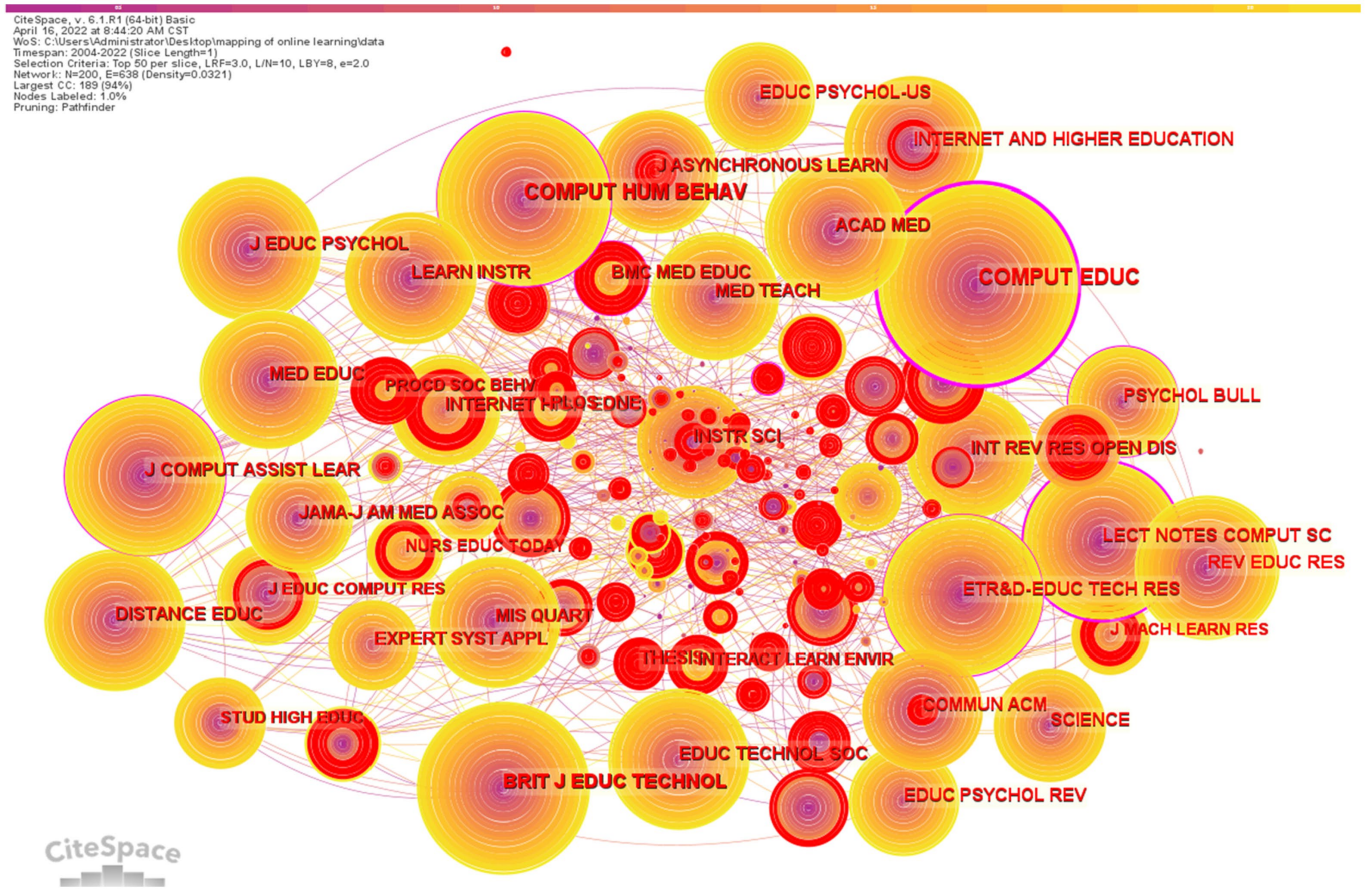
Cited journal network.

To systematically learn about the publication status, the cited journal network is shown in Figure 8 and Table 10. Articles from the Computer and Education have a total citation of 2,684. Articles from Educational Technology Research and Development have a total citation of 1958, and articles from the Internet and Higher Education have a total citation of 1,628. Computers in Human Behavior has a total citation of 1,540. It is clear that these journals are an important source of knowledge in the field of online learning.

**Table 10:** Journals with 600+ citations.

Source	Frequency	Year
*Computers & Education*	2,684	2004
*Educational Technology Research and Development*	1,958	2004
*The Internet and Higher Education*	1,628	2008
*Computers in Human Behavior*	1,540	2004
*British Journal of Educational Technology*	1,371	2004
*Lecture Notes in Computer Science*	1,063	2004
*Educational Technology & Society*	1,060	2006
*Journal of Computer Assisted Learning*	932	2004
*Review of Educational Research*	812	2004
*Journal of Educational Psychology*	700	2004
*Distance Education*	688	2004
*Academic medicine*	672	2004

Nevertheless, for the period (2004–2022), from the perspective of the burst values (Table 11), with a burst value of 126.45, Thesis ranked first. Frontiers in Psychology has a burst value of 82.13. Additional journals with quite high burst values include Education and Information Technologies (77.36), IEEE Access (73.02), American Journal of Distance Education (67.83), PLOS One (63.02), International Journal of Educational Technology in Higher Education (58.35), and Educational Research Review (56.19), which are mainly related to information technology and education. It is clear that these journals are the most active in the field of online learning.

**Table 11:** Top cited journals by citation burst.

Journals	Ranked
*Thesis*	1
*Frontiers in Psychology*	2
*Education and Information Technologies*	3
*IEEE Access*	4
*American Journal of Distance Education*	5
*PLOS One*	6
*International Journal of Educational Technology in Higher Education*	7
*Educational Research Review*	8
*Neurocomputing*	9
*Innovations in Education and Teaching International*	10

### Popular topics and emerging trends

**Figure 9:**
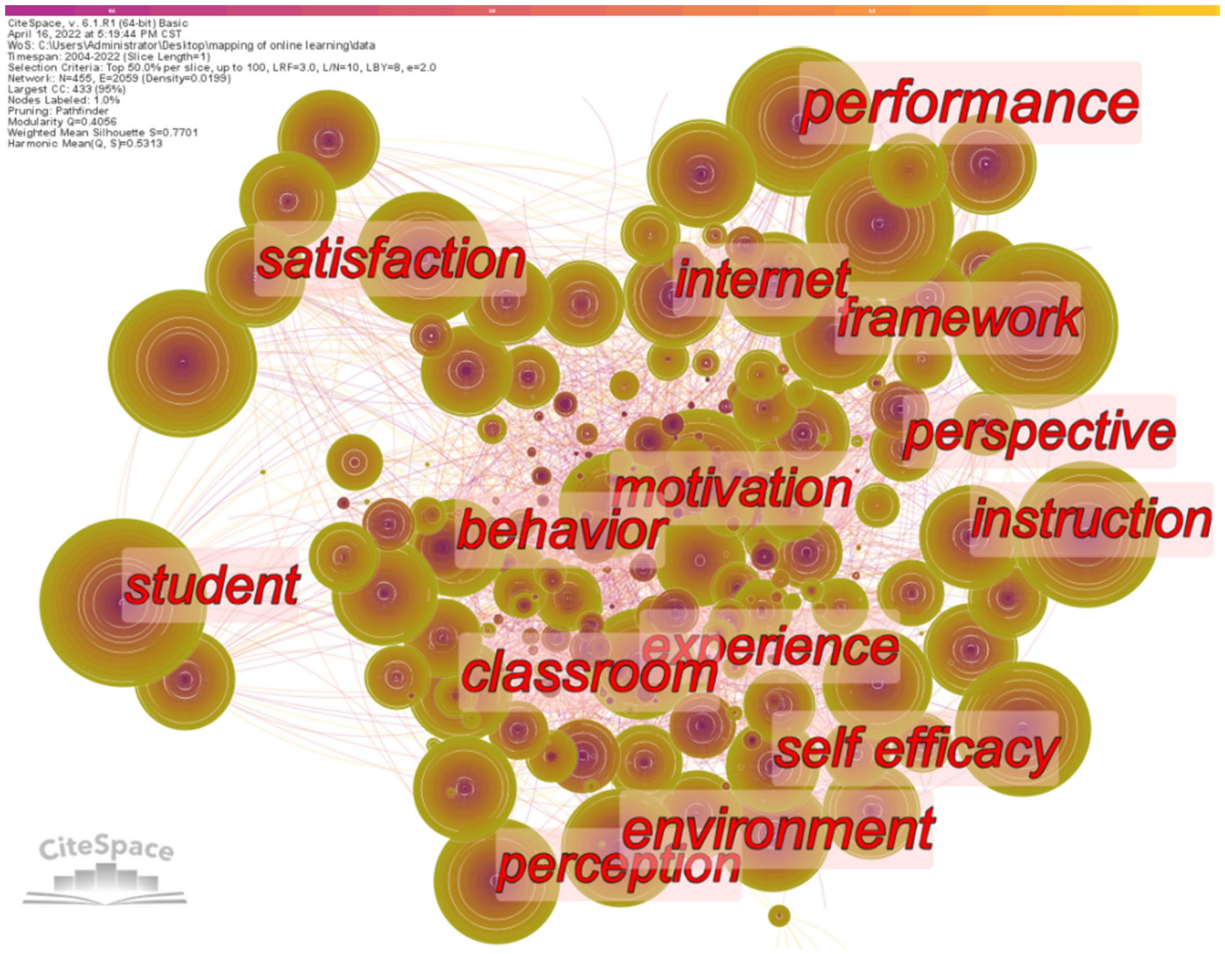
Hot topics.

The findings of the keyword visualization provide insight into popular topics and developing tendencies. Keywords with high frequency are shown in Figure 9 and Table 12, including “student,” “higher education,” “model,” “performance,” “technology,” “impact,” “system,” “design,” “knowledge,” and so on. Table 12 provides a list of the top 24 most frequently used terms in the visualization results to aid in understanding and the highest BC that appeared in the 25,382 articles, and more than 400 occurrences of each term have been indexed. To a significant degree, these keywords might represent current research tendencies and popular subjects.

**Table 12:** Top 24 research keywords by frequency and centrality.

Keywords	Frequency	Keywords	Centrality
student	1,895	performance	0.1
higher education	1,417	algorithm	0.07
model	1,400	student	0.06
performance	1,359	experience	0.06
technology	1,106	professional development	0.06
impact	1,040	higher education	0.05
system	1,017	model	0.05
design	919	system	0.05
knowledge	807	satisfaction	0.05
perception	776	environment	0.05
motivation	685	framework	0.05
satisfaction	572	strategy	0.05
experience	561	information	0.05
environment	555	knowledge	0.05
framework	534	perspective	0.05
strategy	526	web	0.05
skill	469	implementation	0.05
achievement	466	continuing education	0.05
engagement	463	impact	0.04
algorithm	461	quality	0.04
information	456	instruction	0.04
attitude	430	behavior	0.04
quality	422	support	0.04
instruction	403	motivation	0.03

Table 12 shows keywords with both high frequency and high BC, mainly including three types of keywords: student-related, system-related, and teacher-related. First of all, keywords related to students include: “student,” “higher education,” “model,” “performance,” “impact,” “motivation,” “satisfaction,” and “experience.” Most of these studies focus on the construction of models of learning performance, learning motivation, learning satisfaction, learning experience, and other influencing factors of online learning for students in higher education institutions (Wu and Tian, 2021). Rahman et al. (2021) adapted PLS-SEM to study the effects of direct instruction, teacher-learner interaction, learner-learner interaction, and self-efficacy on online learning motivation and satisfaction. The results show that online learning motivation has a significant mediating effect between independent variables and learning satisfaction. In addition, direct instruction, learner-learner interaction, and online self-efficacy significantly predicted students’ online learning satisfaction. Yang et al. (2022) studied students’ online self-evaluation task behavior and its impact on academic performance. Online evaluations after class appear to have a positive impact on students’ exam scores, according to the findings. Nevertheless, despite taking the exams, the learning performance of learners who displayed nonstandard conduct did not necessarily improve.

Secondly, keywords related to learning systems include “system,” “design,” “environment,” “framework,” “algorithm” and “information.” The research related to learning systems mainly focuses on how to design an online learning system to improve the online learning environment and enhance information quality. For example, Buabeng-andoh (2022) studied the influencing factors of learners’ operations of their teaching and learning systems during COVID-19 (Sweller et al., 2007). The results show that nine factors, namely performance expectancy, effort expectancy, attitude, social influence, facilitating condition, self-efficacy, behavioral intention, perceived enjoyment, and system quality, have a significant positive impact on learners’ teaching system operation.

Finally, keywords related to teachers include “strategy,” “quality,” “instruction,” and “knowledge.” Focus on how teachers adopt effective teaching strategies to improve teaching quality and optimize the knowledge transfer process (Wu and Wang, 2018). Hongsuchon et al. (2022) studies the factors influencing the effectiveness of online learning, including students’ self-efficacy, teachers’ self-efficacy, attitudes, technological confidence, educational strategies, and positivity. Learning objectives, according to the findings of this study, might help universities improve the efficacy of students’ online learning by persuading them to enroll in online courses and designing learning techniques that are tailored to their specific requirements.

**Figure 10:**
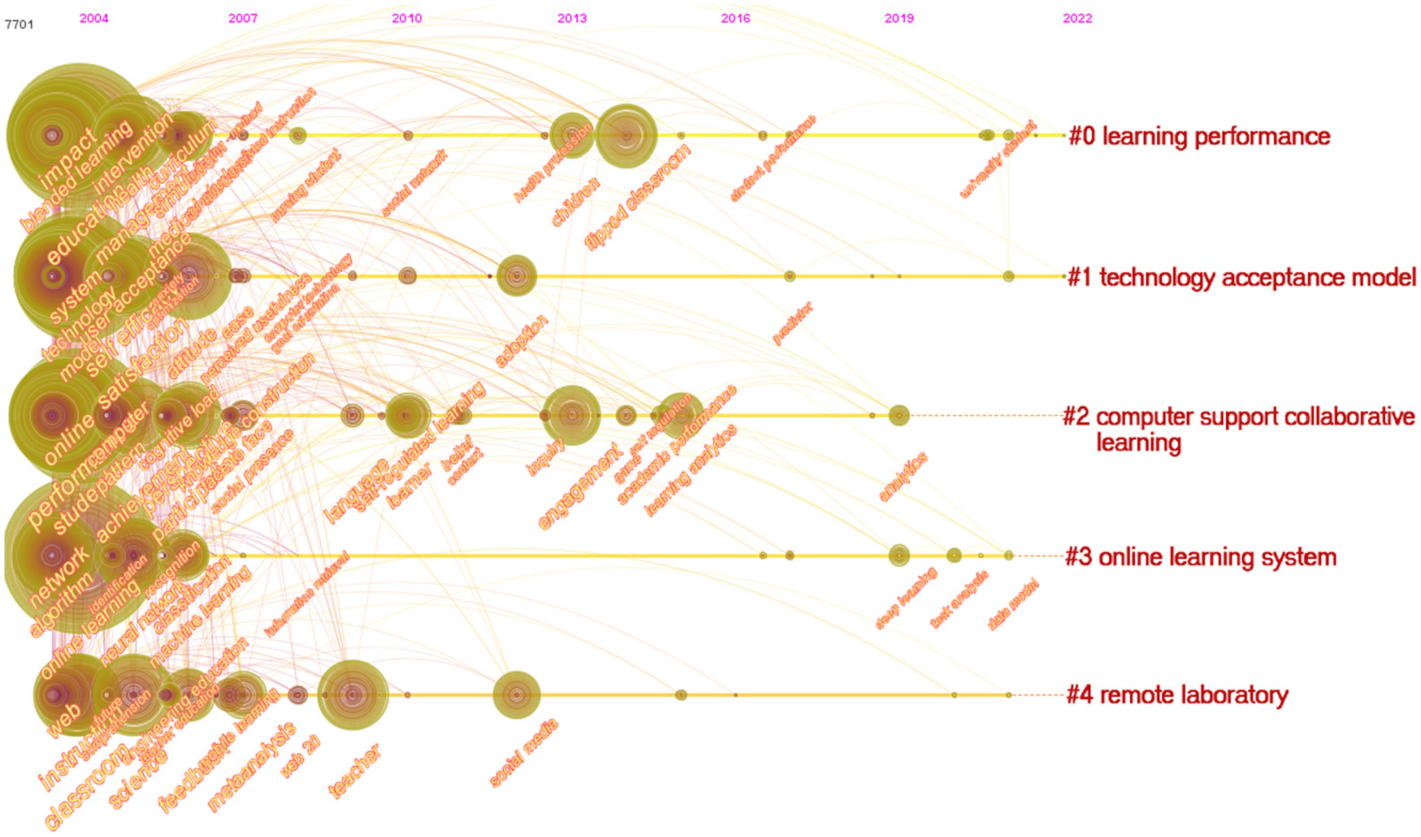
Burst trends of research keywords.

These keyword networks might also be quite useful in identifying revolutionary tendencies in this industry, which would be extremely beneficial. In order to evaluate the developmental route in online learning, a timeline perspective was utilized to assess the tendency of research over the past 20 years, and Figure 10 depicts the changing trend in this field.

Firstly, learning performance, computer support collaborative learning (#2) and online learning system are recently research hotspots. Figure 10 shows that the three clusters have been extensively studied since 2004. Learning performance has been an ongoing research topic in the field of online learning since 2004. As an illustration, for the purpose of categorizing students and making predictions about their future learning outcomes based on characteristics extracted from the recorded data of an online educational system, Behrouz et al. (2004) developed a method known as feature importance mining. A study by Minoru et al. (2012) examined the causal correlations that existed between student traits, note-taking abilities, learning experience, note evaluation, and test results. Meanwhile, Structural Equation Modeling (SEM) was used to track students’ learning processes while they took notes. From an integrated perspective of individuals, environment/technology, and behavior, Wang and Lin (2021) examined the constructs of compatibility, personal innovativeness, convenience, perceived usefulness, continued intention, and healthcare students’ learning performance in online learning system use contexts. This study concluded that personal innovativeness, convenience, and perceived usefulness were the key determinants of students’ learning performance and adoption of an online learning system. Meanwhile, perceived usefulness was the critical mediator between the influences of personal and environmental factors on students’ learning performance. However, this topic received less attention in 2019. With the emergence and spread of COVID-19, countries have had to adopt quarantine measures in order to effectively stop the spread of the virus. As a consequence, a huge amount of pupils have to choose online learning. The topic of learning performance gets researchers’ attention again. For example, Hsiao (2021) investigated the effects of course type and gender on distant learning performance by examining baseline data from the three academic years before COVID-19 (2016–2018) and COVID-19 (2017–2018). The findings indicated that obligatory courses are better suited for distant learning courses, but optional and general education courses are better suited for face-to-face instruction. Males do better in face-to-face courses than females, and there was no discernible difference in their performance between the two teaching approaches (Minaei-Bidgoli et al., 2004). Meanwhile, research on computer support collaborative learning and online learning system are still influencing in the past 10 years. On the one hand, in recent years, computer-supported collaborative learning has mainly focused on inquiry learning, self-regulation learning, and learning analysis. For example, Pietarinen et al. (2021) addressed the real-time shifts in focus and distribution of teachers’ guidance and support of different student groups during in-person computer-supported collaborative inquiry learning in science classrooms. The study concluded that it was discovered that the prior science competency of groups had no influence on teacher supervision and support; rather, instructors guided the groups they regarded to be motivated and eager to collaborate, according to the findings. Wetcho and Na-Songkhla (2021) embedded CSCL into the self-regulating learning process, and explored the predictive effect of self-assessment on task and goal definition. Results indicated that self-evaluation and self-reflection were shown to be somewhat mediated by cooperation. On the other hand, online learning systems mainly focus on system algorithms, neural networks, machine learning, deep learning, task analysis, data models, and so on. For example, based on the neural network algorithm, Peng (2022) developed a method for intelligently teaching English, based on the new algorithm, and the effectiveness of the system for English online learning has been verified. Suparwito et al. (2021) adopted five criteria, namely self-management, personal effort, technology use, self-role recognition, and lecturer role recognition, to analyze students’ views on online learning, and used a random forest algorithm to examine the data. The results showed that the factors affecting students’ satisfaction with online learning included relationships between students and teachers, the adaptation of learning materials to online learning methods, and the use of technology for online learning.

Secondly, Technology Acceptance Model (#1) is still influencing in the past 10 years. Technology acceptance model proposed by Davis (1989) is an important theoretical model to evaluate the use of new technology, including students’ online learning use behavior and satisfactory (Niu and Wu, 2022). This topic mainly discusses the influencing factors of students’ online learning success, use intention, and satisfaction. Figure 10 shows that the external influencing factors of TAM mainly include anxiety, self-efficacy, perceived usefulness, perceived ease of use, etc.For example, based on the technology acceptance model, Hoang et al. (2021) studied the determinants of Vietnamese people’s willingness to borrow and consume credit. Results showed that perceived usefulness played a mediating role in the influence of subjective norms on consumer credit lending intention. Meanwhile, subjective norms also had a significant impact on borrowing intentions. It was worth noting that borrowing intention is unaffected by anxiety or perceived ease of use. On the basis of the technology acceptance model, Hanham et al. (2021) investigated the factors influencing the technology acceptance of preschool teachers. Instructors’ behavioral intentions are high according to the findings, which also demonstrate that perceived usefulness and reported ease of use are significantly predictive of instructors’ behavioral intentions when measured directly. Perceived ease of use and job relevance have a significant effect on perceived usefulness. The impression of external control and computer self-efficacy are the two most important aspects influencing perceived ease of use.

Lastly, of the 25,382 papers and references examined, “remote laboratories” (#4) was the fifth most often cited direction. There is no doubt that the major content areas of this field are engineering education, science, teacher education, mobile learning, and social media. Although there are few studies on remote laboratories in recent years, it is often mentioned in engineering education, science, teacher education, mobile learning, social media and other fields. For example, Lee and Hong (2021) studied science education experts’ perceptions of remote lab sessions during the COVID-19 pandemic by conducting 10 semi-structured interviews with experts in the fields of physics, chemistry, biology, and earth sciences. Those who participated in the Remote Laboratory Sessions were found to have reexamined the purpose and goals of traditional laboratory instruction in light of what they learned. In addition, the study found that students were unable to learn because of a lack of hands-on experience, less contacts between instructors and students, and an increased workload for instructors.

## Discussion and implication

### Discussion

The purpose of this study was to provide a systemic and objective overview of research on online learning. Based on 25,382 documents from 2004 to 2022, collected from the WoS database, Citespace 6.1.R1 (64-bit) was used to undertake an in-depth examination of the research of online learning based on five different perspectives: annual publications, collaboration network (country network, institution network, and author network), co-cited references, cited journals, and co-occurrence analysis of keywords.

With regard to annual publication from 2004 to 2022, the duration of research development is divided into three stages: the slow development stage (2004–2010), the rapid development stage (2011–2016), and the explosion stage (2017–2022). This research result is basically consistent with previous study (Zheng et al., 2019). Zheng et al. (2019) took 20,679 MooCs-related studies in the WoS database from 2012 to 2018 as data sources and concluded that MooCs studies during this period continued to rise, and the growth rate increased year by year. In this study, Due to the COVID-19 pandemic, online learning has become an important learning mode for educational institutions and learners worldwide. Online learning research are developing at an exponential rate as a result.

Cooperative network mainly includes three types: national cooperative network, institutional cooperative network and author cooperative network. The most frequently posted countries were the USA (6948), PEOPLES R CHINA (3396), ENGLAND (1986), SPAN (1921) and AUSTRALIA (1685). The institutions with the highest publications were Open University (231), Nanyang Technology University (202), Monash University (186), the University of Toronto (180), and the University of Sydney (174). HWANG Gwa-Jen (57), HUANG Yueh-min (39), Chen Nian-shing (35), KIRSCHNER PA (34), and KINSHUK (34) have published the most papers. This result is basically consistent with previous studies, but there are some differences, such as the rankings of countries (Li et al., 2021). With WoS and CNKI as data sources (2002–2021) and “online education” and “instructional design” as search keywords, Li searched a total of 670 instructional papers. The results show that the top three core countries are the United States, China, and Canada. According to this study, the reason for the different results lies in the small amount of data in Li′s study. Although it can reflect the major research countries, the statistics on the number of publications in each country are not accurate enough.

Through the cluster analysis of cited references, it is found that online learning research mainly includes seven themes: MOOCs, flipped classroom, COVID-19, computer-supported collaborative learning, technology acceptance model, community of inquiry, and distance learning. The classic literature for each topic is listed in Table 6. In addition, the literature cited frequently in the field of online learning is listed in Table 7. Table 9 specifies the top 9 key pieces of literature in the online learning knowledge map. For cited journals, the most cited journals were: Computer and Education (2,684), Educational Technology Research and Development (1,958), The Internet and Higher Education (1,628), Computer in Human Behavior (1,540), and British Journal of Educational Technology (1,371). Through keyword co-occurrence analysis, high-frequency keywords and high mediating centrality keywords are listed in Table 12. The higher of the above two is the hot spot in online learning research. After in-depth analysis, online learning research hotspots mainly include three categories: student-related, system-related, and teacher-related. Drawing the aforementioned timeline allowed us to group five different subjects together while also revealing the stages at which each topic’s theory and body of knowledge evolved. The results of this study are both identical (Zheng et al., 2019; Li et al., 2021) and different from previous studies (Zhang and Zhao, 2020), including highly cited journals, some research hotspots, and some classical literature. Differences include research topics and future research trends (Hwang et al., 2007). The main reasons for the difference may be as follows: social environment change, research scope, research time span, CiteSpace parameter setting, etc.

During the COVID-19 pandemic, online learning has played an important role in facilitating teaching at higher education institutions. During this period, studies related to online learning mainly have the following characteristics. Firstly, the growth rate is fast. Before 2019, the annual number of articles published was about 1700, while during 2020–2021, the maximum number of articles published per year could reach 4,700 (Figure 2). Second, most studies focus on how to improve students’ academic performance or satisfaction. Due to the sudden arrival of COVID-19, students hardly have any time to adapt, which causes problems such as anxiety, low self-efficacy and lack of interaction (Wu X. and Wang, 2020). In this context, how to improve students’ academic performance or satisfaction with online learning has become the focus of researchers.

### Implication

As for the theoretical implications, based on the bibliometric analysis of literature in the field of online learning from 2004 to 2022, this study generates a comprehensive, clear, and systematic overview of the field, including annual publications, major contributions, research topics and classic literature, current research hotspots, and future research trends.

#### Practical implication

The practical implications of this study mainly include four aspects: Firstly, generating seven research subfields of online learning according to literature co-citation and identifying the classic literature in each subfield can help future researchers identify the classic literature in their respective fields, save literature retrieval time and improve research efficiency. Secondly, according to keyword co-occurrence analysis, the research hot spots and future research trends in online learning are determined. It provides guidance for future researchers to engage in related research. Thirdly, figuring out who are the most important and active people in the field of online learning based on the co-occurrence of their names can help future researchers pay attention to and keep track of their research updates and understand the research trends in this field. Fourthly, the government should encourage online learning platforms to actively participate in the design and development of students’ online learning resources and subsidize them. Meanwhile, the government should formulate relevant policies to regulate the management of students’ online learning platforms to avoid students’ exposure to some bad information.

#### Theoretical implication

First of all, this study provides an overall view of the existing research on online learning, which is conducive to promoting researchers’ comprehensive understanding of this field. Secondly, according to the research content, the existing research is divided into 7 main sub-fields. For example, MOOCs, flipped classroom, covid-19, computer-supported collaborative learning, technology acceptance model, community of inquiry, short learning. It also helps researchers get a complete picture of the field.

## Limitation and future works

CiteSpace was utilized to assess online learning articles from 2004 to 2022, based on the WoS database, in this research. However, despite its excellent accuracy, this method has certain drawbacks. Firstly, due to the limitation of data resources, the earliest data used in this study was in 2004. Therefore, many studies are not included in the collected data set. Furthermore, there are a number of policy and social publications not included in the database, including those from governments or organizations, editorials, and books. This has a great impact on a comprehensive and systematic understanding of the origin and development of online learning. Thus, in future studies, researchers should combine various data sources and develop and extend the research data channels (e.g., SCOPUS, Google Scholar) in order to discuss and evaluate the research issue more extensively and comprehensively. Secondly, this study did not delve into the differences in the development of online learning before and after COVID-19. In the future, a study should be systematically designed, such as a time frame before and after COVID-19, database, retrieval strategies, etc., to compare the laws of change and development trends of online learning between two time frames. Finally, although abundant keywords related to online learning have been selected for retrieval in this study, it is inevitable that some keywords may be omitted. In the future, researchers should enrich retrieval strategies as much as possible and collect more comprehensive and accurate literature to grasp the complete situation of online learning field.

## Data availability statement

The raw data supporting the conclusions of this article will be made available by the authors, without undue reservation.

## Author contributions

YS: conceptualization and writing original draft. YS and LH: data curation. XW: writing—review and editing. All authors contributed to the article and approved the submitted version.

## Funding

This research was supported by the key base project of Science and Technology Project of Education Department of Jiangxi Province in 2018 (No.: GJJ190590); Jiangxi Social Science Planning Project in 2018 (No.: 18JY21); Humanities and Social Science Project of Universities in Jiangxi Province in 2020 (No.: JY20219); Doctoral Research Foundation of Jiangxi Science and Technology Normal University in 2021 (No.: 2021BSQD20); Key Research Base of Humanities and Social Sciences in Universities of Jiangxi Province (No. JD18079); National Social Science Foundation of China 2017 Education General Project (No.: BJA170101); General Project of higher Education and Teaching Reform Research in Hainan Province in 2020: Research on Transformation and Development of Local Undergraduate Colleges in Hainan Province under the Background of Free Trade Zone Construction (No.: HNJG2020-83).

## Conflict of interest

The authors declare that the research was conducted in the absence of any commercial or financial relationships that could be construed as a potential conflict of interest.

## Publisher’s note

All claims expressed in this article are solely those of the authors and do not necessarily represent those of their affiliated organizations, or those of the publisher, the editors and the reviewers. Any product that may be evaluated in this article, or claim that may be made by its manufacturer, is not guaranteed or endorsed by the publisher.

## Footnotes

^1^ The software can be obtained from http://cluster.ischool.drexel.edu/~cchen/citespace/download/ (Retrieved on April 20th, 2022).
